# Interferometric DEM-Assisted High Precision Imaging Method for ArcSAR

**DOI:** 10.3390/s19132921

**Published:** 2019-07-01

**Authors:** Yanping Wang, Yang Song, Yun Lin, Yang Li, Yuan Zhang, Wen Hong

**Affiliations:** 1 School of Information Science and Technology, North China University of Technology (NCUT), Beijing 100144, China; 2 Institute of Electronics, Chinese Academy of Science (IECAS), Beijing 100190, China

**Keywords:** synthetic aperture radar (SAR), ground-based synthetic aperture radar (GBSAR), arc-scanning synthetic aperture radar (ArcSAR), interferometric ArcSAR, DEM assisted SAR imaging

## Abstract

Ground-based arc-scanning synthetic aperture radar (ArcSAR) is the novel ground-based synthetic aperture radar (GBSAR). It scans 360-degree surrounding scenes by the antenna attached to rotating boom. Therefore, compared with linear scanning GBSAR, ArcSAR has larger field of view. Although the feasibility of ArcSAR has been verified in recent years, its imaging algorithm still presents difficulties. The imaging accuracy of ArcSAR is affected by terrain fluctuation. For rotating scanning ArcSAR, even if targets in scenes have the same range and Doppler with antenna, if the heights of targets are different, their range migration will be different. Traditional ArcSAR imaging algorithms achieve imaging on reference plane. The height difference between reference plane and target in scenes will cause the decrease of imaging quality or even image defocusing because the range migration cannot be compensated correctly. For obtaining high-precision ArcSAR image, we propose interferometric DEM (digital elevation model)-assisted high precision imaging method for ArcSAR. The interferometric ArcSAR is utilized to acquire DEM. With the assist of DEM, target in scenes can be imaged on its actual height. In this paper, we analyze the error caused by ArcSAR imaging on reference plane. The method of extracting DEM on ground range for assisted ArcSAR imaging is also given. Besides, DEM accuracy and deformation monitoring accuracy of proposed method are analyzed. The effectiveness of the proposed method was verified by experiments.

## 1. Introduction

Synthetic aperture radar (SAR) is capable of high-resolution imaging all-day and all-weather conditions [1,2]. As a complex image, the SAR image contains amplitude and phase information. We can get the deformation of the scenes by differential interferometric SAR (D-InSAR). Therefore, SAR is widely used in the field of ground deformation monitoring [3,4,5]. Especially the spaceborne SAR can achieve ground deformation monitoring in a wide range of scenes [6]. However, the spaceborne SAR systems and airborne SAR system require a long revisit cycle. They cannot realize continuous and repeated monitoring of a region. As an alternative, the GBSAR can achieve continuous and repeated monitoring of a region and feedback monitoring information in real time. In view of the above advantages, GBSAR has become one of the important means for deformation monitoring of dams’ walls, buildings and slope [7,8,9,10]. 

The conventional GBSAR system scans the scenes along the linear rail. Its synthetic aperture is generated by moving the radar on the rail [11]. This type of working mode limits its field of view. A new mode GBSAR called ArcSAR can solve this problem. The ArcSAR system scans the surrounding scenes by the antenna attached to the rotating boom which extending from the center of the rotating platform. Its synthetic aperture is generated by the rotation of the antenna [12]. Therefore, under the premise of ensuring the resolution of the system, ArcSAR can cover the 360-degree scenes in once scanning, which effectively improve the field of view. Currently, several teams are working on the ArcSAR system [12,13,14,15,16,17,18,19,20], and the imaging algorithms for ArcSAR are also proposed.

Lee et al. deduced the geometric model and signal model of the ArcSAR system and proposed the imaging algorithm for ArcSAR [17,18]. Luo Yunhua et al. proposed the fast imaging algorithm for ArcSAR and used differential interferometric ArcSAR for ground deformation monitoring [19,20]. However, the above ArcSAR imaging algorithms perform the imaging on the reference plane. The imaging accuracy of the ArcSAR system is affected by the terrain fluctuation. For rotating scanning ArcSAR system, even if the targets in the scenes have the same range and Doppler with the antenna, the targets with different height have different range migration. Therefore, if the height of reference plane is inconsistent with the actual height of target in the scenes, the height difference between the reference plane and the target will cause the decrease of imaging quality or even image defocusing because the range migration cannot be compensated correctly.

To acquire the high precision ArcSAR image, the DEM of the scenes can be used to assist ArcSAR imaging. In this paper, we propose an interferometric DEM-assisted high precision imaging method for ArcSAR. Firstly, the DEM image of the scenes is acquired by interference with the ArcSAR slant range images. The acquired DEM image is on the slant range. However, we require the DEM image on the ground range to assist ArcSAR imaging. Thus, we next transform the DEM image on the slant range to the ground range. Finally, with the assist of the DEM image on the ground range, the target in the scenes can be imaged on its actual height. The proposed method does not rely on external DEM data. It can effectively avoid the decrease of ArcSAR imaging quality. 

This paper proceeds as follows. The ArcSAR geometric model and signal model that consider the terrain of the scenes is introduced in Section 2. The error caused by ArcSAR imaging on the reference plane is analyzed in Section 3. The principle of the interferometric DEM-assisted high precision imaging method is given in Section 4. The DEM accuracy and deformation monitoring accuracy of proposed method are analyzed in Section 5. The effectiveness of the proposed high precision imaging method is verified by experiment in Section 6. In Section 7, we discuss the concluding remarks.

## 2. The Geometric Model and Signal Model of ArcSAR

**Figure 1:**
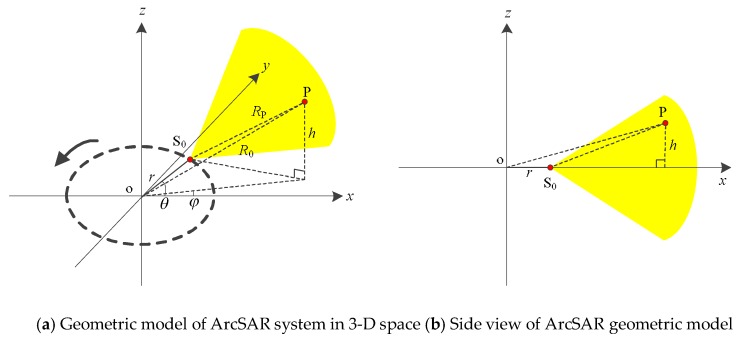

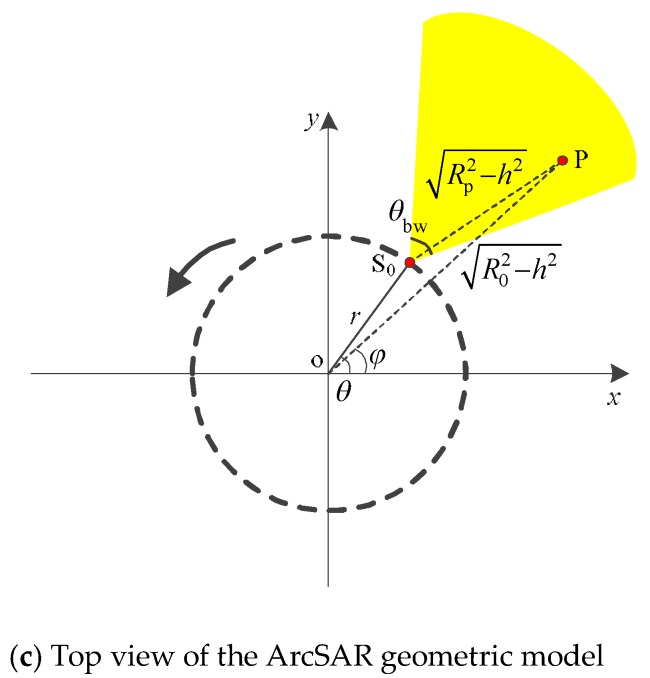
The geometric model of ArcSAR system.

Figure 1 shows the geometric model of ArcSAR system. The antenna of ArcSAR mounted by the boom rotates counterclockwise. It transmits and receives signals at equal intervals. The coordinate *z*-axis is the rotation axis of the ArcSAR system. We select *x*o*y* plane as the rotation plane. Point o is the rotation center. Point P represents the target in the scenes. Its coordinates can be expressed as:

(1) $$\left(\sqrt{R_{0}^{2}-h^{2}}\cos\varphi ,\sqrt{R_{0}^{2}-h^{2}}\sin\varphi ,h\right)$$

where *R*_0_ is the distance between the target P and the point o. *h* is the height of the target P. *φ* stands for the azimuth angle of the target position. Point S_0_ is the position of the antenna phase center (APC). Its coordinates are

(2) (rcosθ,rsinθ,h)

where *r* represents the length of the boom and *θ* is the rotation angle of the boom. 

Taking example for linear frequency modulation (LFM) signal, the echo signal of the target P in ArcSAR system is shown as:

(3) $$S\left(\theta ,t_{r}\right)=\delta_{p}rect\left(\left(t_{r}-2R_{p}/c\right)/T_{p}\right)\cdot \mathrm{rect}(\left(\theta -\varphi \right)/\theta_{\mathrm{bw}})\;\exp\left(j\pi K_{r}{\left(t_{r}-2R_{p}/c\right)}^{2}\right)\cdot \exp\left(-j4\pi f_{c}R_{p}/c\right)$$

where *δ*_p_ is the backscattering coefficient, *t*_r_ is the fast time of the ArcSAR system, *c* represents the speed of light, *T*_p_ stands for the signal pulse width, *θ*_bw_ is the antenna beam width, *K*_r_ represents the linear frequency modulation (LFM) rate, and *f*_c_ is the center frequency. *R*_p_ represents the distance between target P and the APC, which can be expressed as:

(4) $$R_{p}=\sqrt{R_{0}^{2}+r^{2}-2r\sqrt{R_{0}^{2}-h^{2}}\cos(\theta -\varphi )}$$

We perform pulse compression of *S*(*θ*,*t*_r_) in the frequency domain on the range direction. The result can be expressed as:

(5) $$S(\theta ,f)=\delta_{p}\cdot \mathrm{rect}(\left(\theta -\varphi \right)/\theta_{\mathrm{bw}})\cdot \mathrm{rect}(f/B_{r})\cdot \exp[-j4\pi (f+f_{c})R_{p}/c]$$

where *f* is the current frequency in frequency domain after the spectral transformation of signal *S*(*θ*,*t*_r_), *B*_r_ is the bandwidth. Since the echo signal has a shift-invariant characteristic in the azimuth direction, targets with different *φ* have the same form of range migration if they are the same distance from the rotation center. Therefore, for the convenience of derivation, in this paper, we assume φ = 0. The backscattering coefficient is also neglected. *R*_p_ and *S*(*θ*, *f*) can be rewritten as a current frequency in a frequency domain:

(6) $$R_{p}=\sqrt{R_{0}^{2}+r^{2}-2r\sqrt{R_{0}^{2}-h^{2}}\cos\theta }$$

(7) $$S(\theta ,f)=\mathrm{rect}(\theta /\theta_{\mathrm{bw}})\cdot \mathrm{rect}(f/B_{r})\cdot \exp[-j4\pi (f+f_{c})R_{p}/c]$$

## 3. Analyzing the Error Caused by ArcSAR Imaging on the Reference Plane

In this section, the error model of ArcSAR imaging on the reference plane is first built. Then, the height difference threshold for determining whether the ArcSAR image will severely defocus is calculated. We also explore the relationship between height difference threshold and system parameters. 

### 3.1. The Error Model of ArcSAR Imaging on the Reference Plane

**Figure 2:**
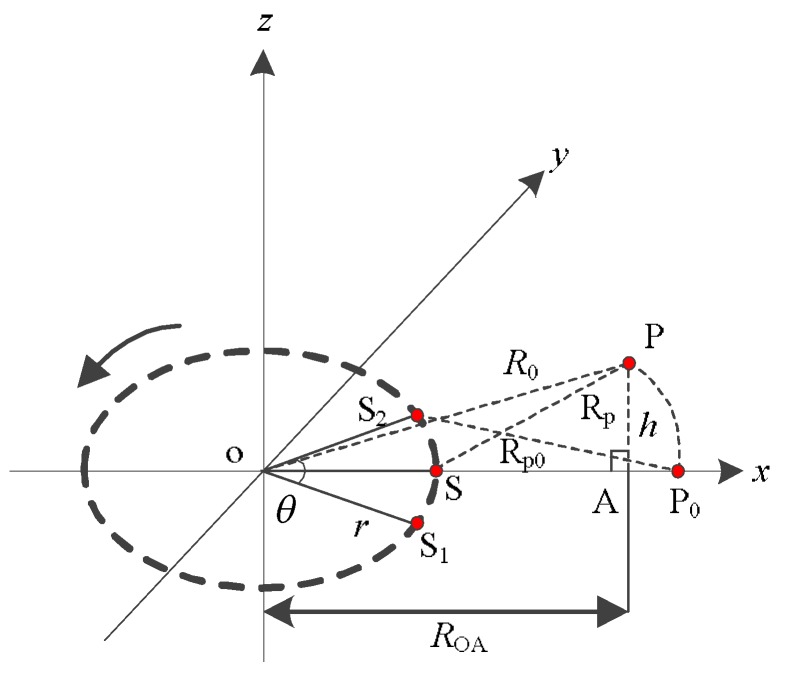
Target P and its imaging result on the reference plane.

As shown in Figure 2, the antenna rotates counterclockwise from S_1_ to S_2_. When the antenna rotates to S, it is closest to the target P. The *x*o*y* plane is defined as the reference plane (the *x*o*y* plane is defined as the reference plane in subsequent parts of this paper). We use the backward projection algorithm (BP algorithm) to achieve the imaging of the target P on the reference plane. The P_0_ is the imaging result on the reference plane. It has the same range and Doppler from the antenna with target P. However, referring to the geometric model given in Figure 2, target P and P_0_ has different range migration because the height of the reference plane is inconsistent with the actual height of the target P. 

The inverse Fourier transform is performed on *S*(*θ*, *f*) at the range direction to obtain its time domain form, which can be expressed as:

(8) $$S_{c}(\theta ,t_{r})=\sin c\left(B_{r}\left(t_{r}-2R_{p}/c\right)\right)\exp\left(-j\frac{4\pi f_{c}}{c}R_{p}\right)$$

The exponential term of *S*_c_(*θ*,*t*_r_) represents the range migration, which must be compensated during the process of focusing. Therefore, the matched filter is used to compensate for the range migration of *S*_c_(*θ*,*t*_r_). The ideal matched filter is expressed as follows:

(9) $$H(\theta ,t_{r})=\exp\left\{j\frac{4\pi f_{c}}{c}\cdot R_{p}\right\}$$

However, the actual matched filter used to compensate for the range migration of target P is different from the ideal matched filter because target P and P_0_ have different range migrations. The actual matched filter contains the phase error, which means the range migration of target P cannot be compensated correctly. The actual matched filter can be expressed as:

(10) $$H(\theta ,t_{r})=\exp\left\{j\left(\frac{4\pi f_{c}}{c}\cdot R_{p}+\Delta p\right)\right\}$$

where ∆*p* represents the phase error:

(11) $$\Delta p=\frac{4\pi f_{c}}{c}\cdot (R_{p0}-R_{p})$$

where *R*_p0_ represents the distance between P_0_ and the APC, which can be expressed as:

(12) $$R_{p0}=\sqrt{{\left(R_{\mathrm{OA}}-r\right)}^{2}+r^{2}+h^{2}-2r\sqrt{{\left(R_{\mathrm{OA}}-r\right)}^{2}+h^{2}}\cos\theta }$$

(13) $$R_{\mathrm{OA}}=\sqrt{R_{0}^{2}-h^{2}}$$

where *R*_OA_ is the ground range from the target to the rotation center. Therefore, the imaging result of the target P on the reference plane is

(14) $$G_{p0}=\int_{\theta }S_{c}(\theta ,t_{r})\cdot H(\theta ,t_{r})d\theta =\int_{\theta }B_{r}\sin c\left(B_{r}\left(t_{r}-2R_{p}/c\right)\right)\exp\left(j\Delta p\right)d\theta$$

The matched filter in Equation (10) cannot correctly compensate for the range migration. Thus, the imaging quality of the target P on the reference plane will decrease. 

Then, we derive the condition for the defocusing of the ArcSAR imaging result on the reference plane. According to Equation (11), the occurrence of ∆*p* is due to the slant range error caused by *R*_p_ and *R*_p0_. We define the slant range error as the ∆*R*, which can be expressed as:

(15) $$\Delta R=R_{p0}-R_{p}$$

**Figure 3:**
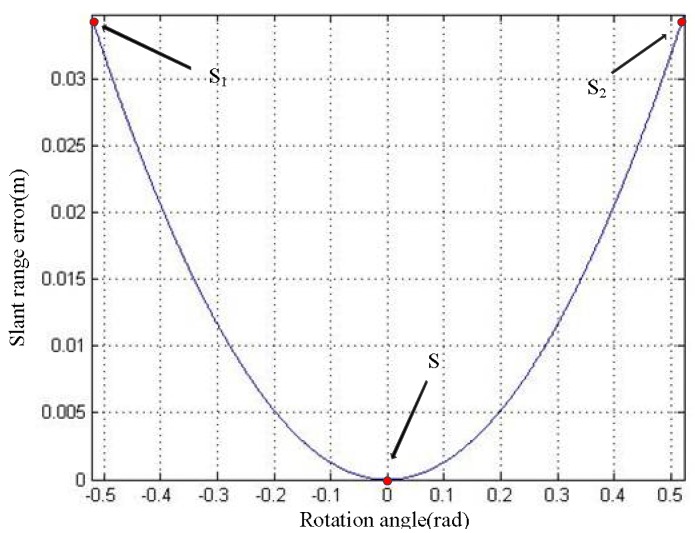
The *θ*–∆*R* relationship curve.

Equations (6) and (12) indicate that the ∆*R* is the function of *θ*. The relationship curve of *θ* and ∆*R* is shown in Figure 3. S_1_, S and S_2_ are marked in the curve. As can be seen from the curve, when the antenna is at point S, the value of slant range error is zero. As the antenna deviates from position S, the value of slant range error gradually increases. When the antenna is at S_1_ and S_2_, the value of slant range error is the largest. We define the maximum slant range error as ∆*R*_max_.

The maximum phase error acceptable for SAR image is π/4 [21]. If the phase error exceeds π/4, the SAR image will severely defocus (when the phase error is less than π/4, this error is too small for SAR imaging to be ignored). Therefore, the condition for severe defocusing of the ArcSAR imaging result on the reference plane is:

(16) $$\begin{array}{c} \Delta p_{\max}\geq \pi /4\\ \frac{4\pi f_{c}}{c}\cdot \Delta R_{\max}\geq \pi /4\\ \Delta R_{\max}\geq \frac{\lambda }{16} \end{array}$$

where *λ* represents the wavelength.

### 3.2. The Relationship between Height Difference Threshold and System Parameters 

In this part, we calculate the height difference threshold for determining whether the ArcSAR imaging result on the reference plane will severely defocus, and analyze the relationship between the height difference threshold and the system parameters. We define the height of the reference plane as *h*_ref_, which the value is 0 m. The geometric relationship is shown in Figure 2. The necessary parameters are given in Table 1.

**Figure 4:**
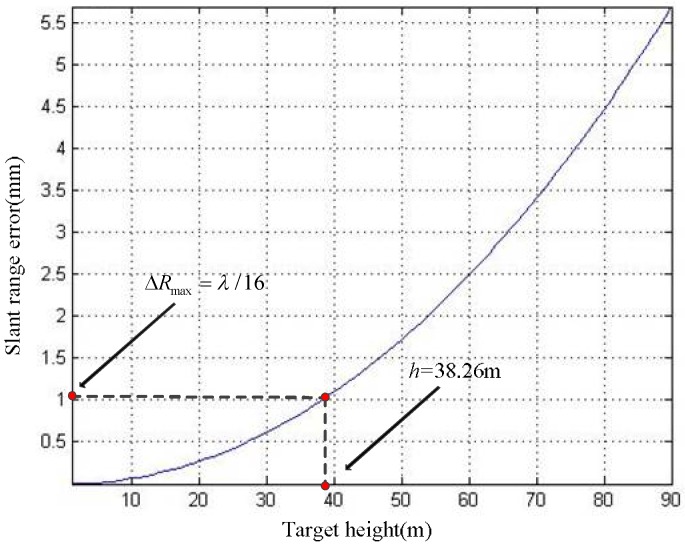
The *h*–∆*R*_max_ relationship curve.

*R*_sp_ is the minimum slant range between the antenna and the target during the rotation. The relationship between height of the target and ∆*R*_max_ is as follows:

(17) $$R_{p0\max}-R_{p\max}=\Delta R_{\max}$$

(18) $$R_{p0\max}=\sqrt{{\left(r\sin\frac{\theta }{2}\right)}^{2}+{\left(R_{\mathrm{sp}}+r-r\cos\frac{\theta }{2}\right)}^{2}}$$

(19) $$R_{p\max}=\sqrt{{\left(r\sin\frac{\theta }{2}\right)}^{2}+{\left(\sqrt{R_{\mathrm{sp}}-h^{2}}+r-r\cos\frac{\theta }{2}\right)}^{2}+h^{2}}$$

where *R*_pmax_ is the maximum slant range between the antenna and the target P during system rotation, *R*_p0max_ represents the maximum slant range between the antenna and P_0_ during system rotation. According to Equations (17)–(19) and the parameters given in Table 1, we can get the *h*–∆*R*_max_ relationship curve (Figure 4).

**Figure 5:**
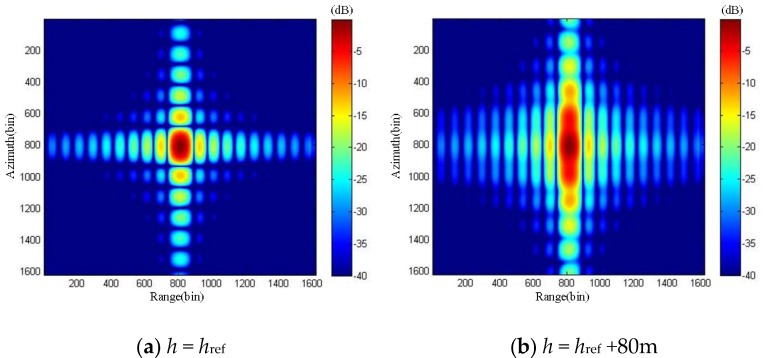
Imaging simulation results of target P on the reference plane.

With reference to Figure 4, it can be seen that the ∆*R*_max_ increases with *h*. Applying the parameters of Table 1 to Equations (17)–(19), we calculate that the height difference threshold is 38.26 m. When *h* exceeds 38.26 m, the ArcSAR image of target P on the reference plane will severely defocus. We give the simulated imaging results of the target P on the reference plane in Figure 5. When *h* is 0 m, the imaging result of target P on the reference plane has no defocusing because the height of the reference plane is consistent with the actual height of the target at this time. When *h* is 80 m, the height of the target P exceeds height difference threshold, thus the imaging result of target P on the reference plane appears severe defocusing.

The height difference threshold is determined by the system parameters given in Table 1. Therefore, the change of system parameters has an impact on height difference threshold: under the premise of changing only single system parameter, the increase of *θ*_bw_, *θ* and *R*_sp_ will cause the height difference threshold to rise, while the increase of the *f*_c_, *r* will cause the height difference threshold to decrease. 

## 4. The Principle of Interferometric DEM-Assisted High Precision Imaging Method for ArcSAR

From the analysis of previous section, the ArcSAR imaging result on the reference plane is affected by the terrain fluctuation. For acquiring high precision ArcSAR image, an interferometric DEM-assisted high precision imaging method for ArcSAR is proposed in this paper. The interferometric ArcSAR is utilized to acquire the scenes DEM on the slant range. Since DEM-assisted ArcSAR imaging requires the DEM image on the ground range, we propose a polar coordinate transformation method for transforming DEM image from slant range to ground range. With the assist of DEM image on the ground range, the target in the scenes can be imaged on its actual height.

**Figure 6:**
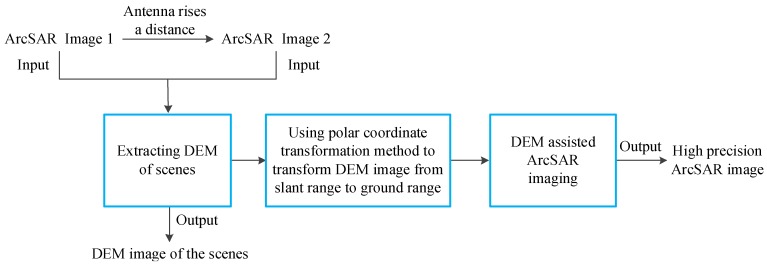
Flow chart of the proposed high precision ArcSAR imaging method.

The steps of the above method are as follows: (1) The ArcSAR system is utilized to scan the same scenes at two different heights for getting ArcSAR Image 1 and ArcSAR Image 2. It should be noted that the acquired two ArcSAR images are the imaging results on the reference plane. Although the imaging accuracy of them is affected by the terrain fluctuation, their phase information can be used to obtain the interferometric phase. (2) ArcSAR Image 1 and ArcSAR Image 2 are used for interference to obtain interferometric phase. (3) We perform the operations of flat phase removing and phase unwrapping for the interferometric phase. (4) The unwrapped interferometric phase is used to inverse the DEM of the scenes. (5) The DEM image in slant range is transformed to the ground range by proposed polar coordinate transformation method. (6) The DEM of the scenes is used to assist ArcSAR imaging. A flow chart of the above method is shown in Figure 6.

### 4.1. Interferometric ArcSAR Extraction DEM of Scenes

Since the method of extracting DEM of the scenes using InSAR is relatively mature [22,23], we only briefly describe the main principle of interferometric ArcSAR in this section.

**Figure 7:**
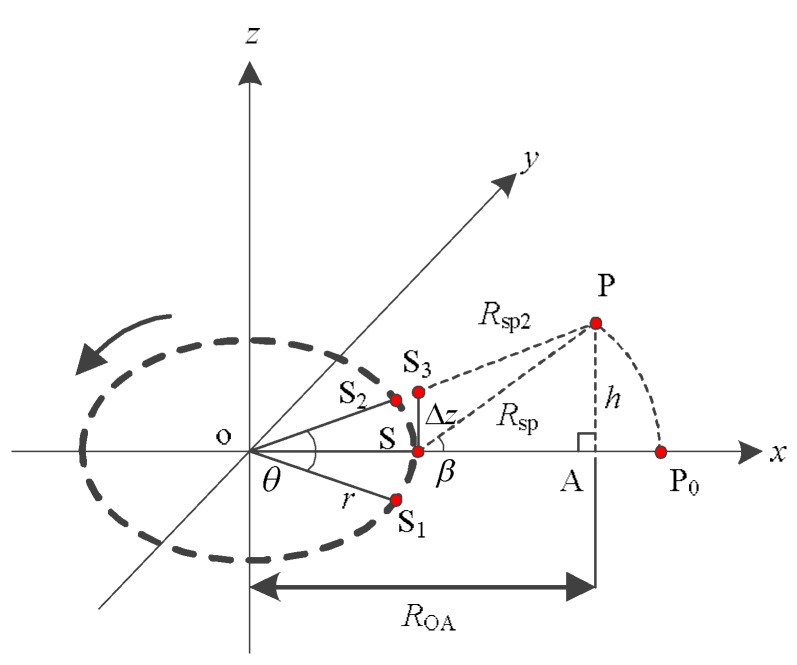
The model of interferometric ArcSAR.

The model of interferometric ArcSAR is shown in Figure 7. Firstly, the antenna scans the target P at two different heights to obtain the imaging results on the reference plane, which can be shown as:

(20) $$G_{1}=E_{s}\exp\left(-j\frac{4\pi }{\lambda }R_{\mathrm{sp}}\right)$$

(21) $$G_{2}=E_{s}\exp\left(-j\frac{4\pi }{\lambda }R_{\mathrm{sp}2}\right)$$

where G_1_ is the ArcSAR image taken when antenna is rotating on the *x*o*y* plane and G_2_ is the ArcSAR image taken after raising the antenna to leave the *x*o*y* plane. The height baseline is ∆*z*. *E*_s_ stands for the amplitude information of the SAR image. *R*_sp2_ represents the distance from S_3_ to the target P.

Then, we can acquire the complex conjugate result of G_1_and G_2_:

(22) $$G_{2}G_{1}^{*}=E_{s}^{2}\exp\left(-j\frac{4\pi }{\lambda }R_{\mathrm{sp}2}\right)\cdot \exp\left(j\frac{4\pi }{\lambda }R_{\mathrm{sp}}\right)=E_{s}^{2}\exp\left(j\frac{4\pi }{\lambda }\left(R_{\mathrm{sp}}-R_{\mathrm{sp}2}\right)\right)$$

According to Equation (22), the interferometric phase *p*_1_ can be expressed as:

(23) $$p_{1}=\frac{4\pi }{\lambda }\left(R_{\mathrm{sp}}-R_{\mathrm{sp}2}\right)$$

Based on the work in [22], plane wave approximation is used to simplify the derivation process. Thus, *R*_sp2_ can be approximated as:

(24) $$R_{\mathrm{sp}2}=R_{\mathrm{sp}}-\delta$$

where δ is the difference of *R*_sp_ and *R*_sp2_, which can be expressed as

(25) $$\delta =\Delta z\frac{h}{R_{\mathrm{sp}}}=\Delta z\sin\beta$$

Thus, the *p*_1_ is rewritten as:

(26) $$p_{1}=\frac{4\pi }{\lambda }\Delta z\sin\beta$$

*β* is the angle between *R*_sp_ and the reference plane, which can be expressed as:

(27) $$\beta =\sin^{-1}\left(\frac{\lambda }{4\pi \Delta z}p_{1}\right)$$

The height of the target P can be expressed as:

(28) $$h=R_{\mathrm{sp}}\sin\beta =R_{\mathrm{sp}}\left(\frac{\lambda }{4\pi \Delta z}p_{1}\right)$$

### 4.2. DEM Image Transforming from Slant Range to Ground Range

In this paper, the ArcSAR images used for extracting DEM are the slant range images. Therefore, on the range direction, the pixel units of the ArcSAR image are equally spaced according to the slant range. The DEM image obtained by the interference with the ArcSAR images is also the slant range image (each pixel unit on the DEM image represents DEM data). However, assisted ArcSAR imaging requires the use of DEM image on the ground range. Thus, it is necessary to transform the DEM image from slant range to ground range.

The process of DEM image from slant range to ground range in linear scanning GBSAR is generally achieved in Cartesian coordinate system. This is because the range direction and azimuth direction of the linear scanning GBSAR are along the coordinate axes of the Cartesian coordinate. Thus, in the Cartesian coordinate system, the linear scanning of GBSAR only requires operating in one dimension to complete the transformation of the DEM image. 

However, if we realize the transformation of ArcSAR DEM image in Cartesian coordinate system, we have to operate in two dimensions. Because the azimuth direction of the ArcSAR system is the rotation direction of the antenna, and the range distance of the ArcSAR is the radial direction of the antenna motion track. To simplify the transformation process of ArcSAR DEM image, we propose a transformation method in polar coordinate system. 

**Figure 8:**
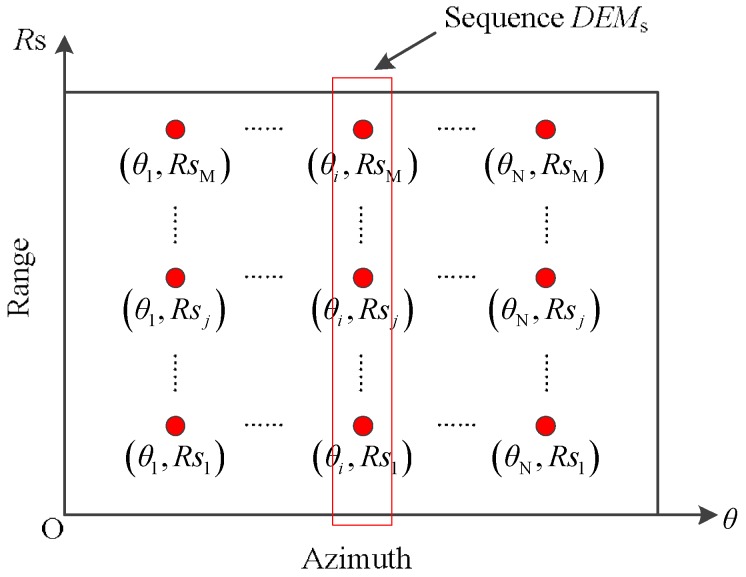
The DEM image in the polar coordinate system.

**Figure 9:**
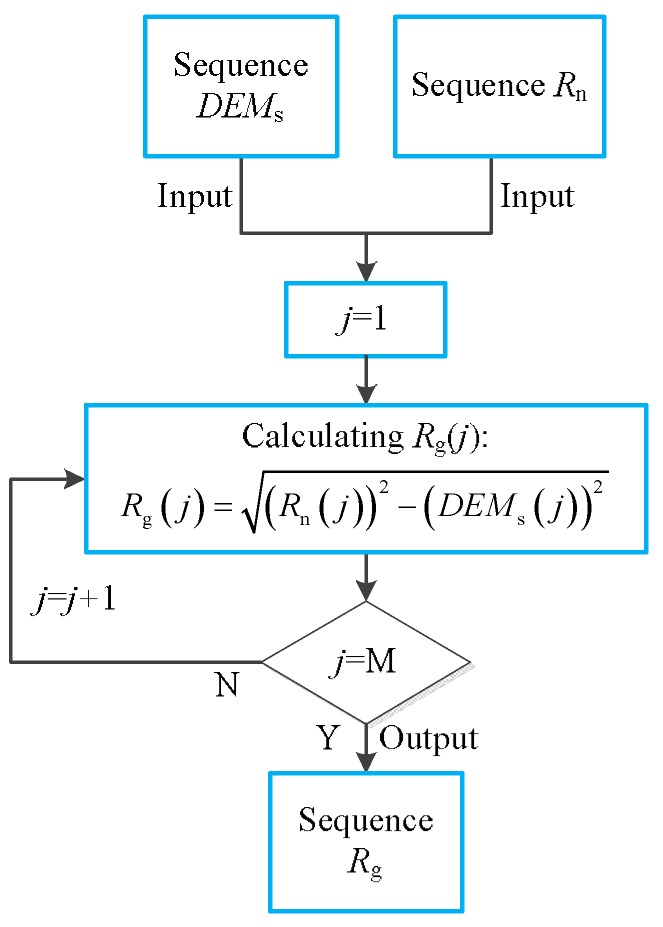
Flow chart of calculating the sequence *R*_g._

The DEM image in polar coordinate system is shown in Figure 8. The *θ*-axis of the coordinate system represents the azimuth direction. The pixel units in azimuth direction are equally spaced according to the rotation angle (*θ*) of the ArcSAR system, and there are N pixel units in this direction. The *Rs*-axis of the coordinate system represents the range direction. The pixel units in this direction are equally spaced according to the slant range (*Rs*), and there are M pixel units in this direction. The size of the DEM image is M×N. The coordinates of the pixel units are also indicated in Figure 8. Taking the pixel unit with coordinate (*θ_i_*, *Rs_j_*) as an example, we discuss the process of transforming the pixel unit from slant range to ground range. The specific operation steps of the proposed transformation method are as follows,

1. When *θ* = *θ_i_* (*i* = 1, 2, ..., N), the sequence *DEMs* are taken from the DEM image. This sequence stores the DEM data in the DEM image at *θ* = *θ_i_*. Its size is M×1. At the same time, we define a slant range sequence *R*_n_. It stores the slant range corresponding to each element in the sequence *DEMs*, which can be expressed as:
  (29) $$R_{n}=\left[Rs_{1},Rs_{2},...,Rs_{j},...,Rs_{M}\right]\left(j=1,2,...,M\right)$$
2. We calculate the ground range sequence *R*_g_ using the geometric relationship between the sequence *DEMs* and the sequence *R*_n_. The flow chart of the calculation process is shown in Figure 9. Sequence *R*_g_ stores the ground range corresponding to each DEM data in the sequence *DEMs*.
3. We define a ground range sequence *R*_ge_, which is an increasing sequence. Its size is M × 1. Furthermore, the largest element of this sequence is *R*_ge_(M), which is equal to the *R*_g_(M). The difference of adjacent elements in *R*_ge_ is fixed.
4. We acquire the element in the sequence *R*_g_ that is numerically closest to the element *R*_ge_ (*j*), which we define as *R*_ne_.
5. According to the position of *R*_ne_ in the sequence *R*_g_, the DEM data corresponding to the element *R*_ne_ in the sequence *DEMs* can be found. We named these DEM data as *H*_ne_.
6. Since *R*_ge_(*j*) is numerically close to *R*_ne_, the position of their corresponding DEM data on the DEM image will be very close. Therefore, we assume that the DEM data of *R*_ge_(*j*) are also *H*_ne_.
7. According to *R*_ge_(*j*) and *H*_ne_, the slant range corresponding to *R*_ge_(*j*) can be calculated:
  (30) $$R_{\mathrm{new}}=\sqrt{R_{\mathrm{ge}}^{2}\left(j\right)+H_{\mathrm{ne}}^{2}}$$
8. Let *R*_new_ interpolate on the sequence *R*_n_ (the purpose of this operation is to find the position of *R*_new_ on the sequence *R*_n_). According to the position of *R*_new_ on the sequence *R*_n_, we can find the DEM data corresponding to *R*_new_ on the sequence *DEMs*, which we define as *H*_new_.
9. Since *R*_ge_(*j*) is the ground range corresponding to *R*_new_, the DEM data of *R*_ge_(*j*) are also *H*_new_.

**Figure 10:**
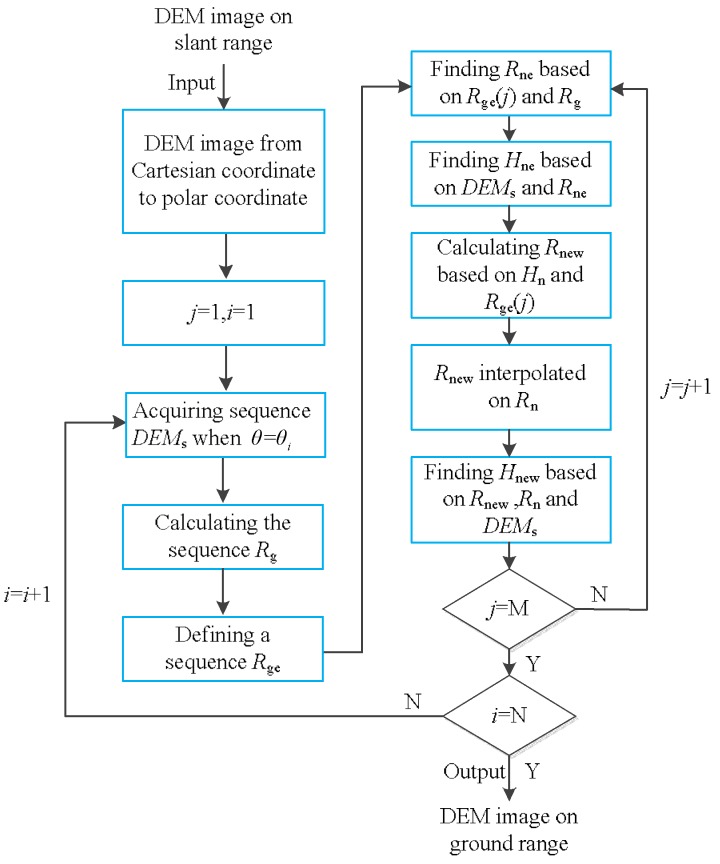
The polar coordinate transformation method proposed in this paper.

Through the above steps, the DEM data with coordinate (*θ_i_*, *Rs_j_*) on the slant range are transformed to the ground range. The coordinate of these DEM data on the ground range is (*θ_i_*, *R*_ge_(*j*)). The above steps only describe the process of transforming one DEM datum in the DEM image from the slant range to ground range. The complete transformation process is shown in Figure 10.

## 5. Accuracy Analysis

We extract the DEM using the SAR images imaged on the reference plane. The defocusing of SAR image causes the larger phase error, which has the impact on the accuracy of the DEM and further affects the imaging quality. The defocusing can cause two types of phase error. The first is the random phase error due to the decrease of the SAR image’s signal-to-noise ratio (SNR). The second is the phase error due to the difference in phase distortion of the SAR image pair used for the interference. In this section, we analyze the impact of the above two phase errors on DEM accuracy. Since the application background of the interferometric ArcSAR in this paper is deformation monitoring, the deformation monitoring accuracy is also analyzed. 

In addition, it should be noted that the purpose of analyzing DEM accuracy and deformation monitoring accuracy is to demonstrate the effect of DEM assist on improving the accuracy of the results. In practice, the analysis of DEM accuracy and deformation monitoring accuracy also needs to consider more complex factors such as baseline decoherence and time decoherence. Since these factors are not relevant to our topic, they are not considered in this paper.

### 5.1. The DEM Accuracy Analysis

#### 5.1.1. Phase Error Analysis as the Decrease of the SNR Caused by Image Defocusing

Defocusing causes the decrease of the SAR image’s SNR, which results in larger phase error. We analyze this type of phase error in this section. The SNR loss of the imaging result is defined as *S*_loss_. According to the geometric model in Figure 7, we apply the numerical analysis method to obtain the *h*–*S*_loss_ curve of the imaging result P_0_. The relationship between *h* and *S*_loss_ can be expressed as:

(31) $$S_{\mathrm{loss}}=10\mathrm{lg}{\left(\left|\frac{\sum_{i=-\frac{\theta }{2}}^{i=\frac{\theta }{2}}\exp\left(j\frac{4\pi f}{c}\Delta R\left(i,h\right)\right)}{\theta }\right|\right)}^{2}$$

**Figure 11:**
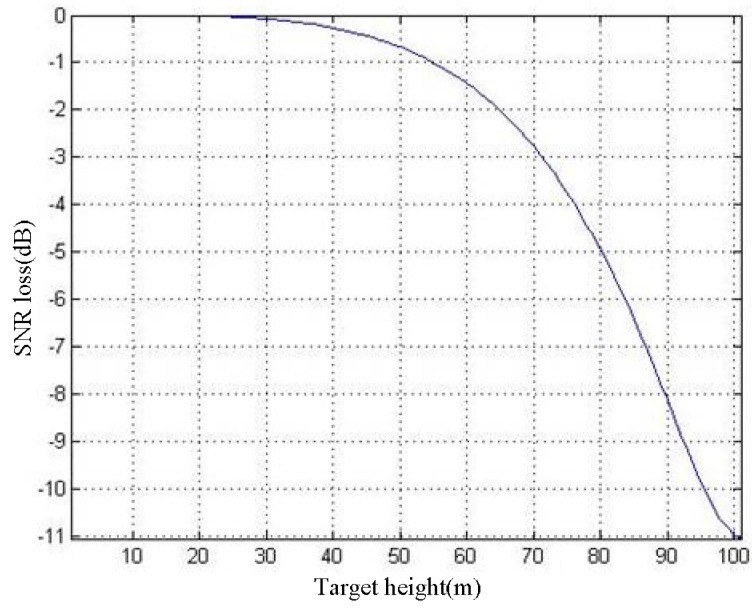
*h–S*_loss_*curve*.

The necessary parameters are given in Table 1. The *h*–*S*_loss_ curve is shown in Figure 11. 

It can be seen in Figure 11 that, as *h* increases, the SNR loss gradually becomes severe. We assume that the SNR of the imaging result is 20 dB when *h* = 0 m. The SNR will decrease to 9.03 dB when h = 100 m, which means that the imaging result is seriously defocused.

According to the authors of [24], the root mean square error *σ*_ps_ of the phase error due to the decrease of the SNR can be expressed as:

(32) $$\sigma_{\mathrm{ps}}=\frac{1}{\sqrt{2NL}}\cdot \frac{\sqrt{1-\rho^{2}}}{\rho }$$

where *NL* stands for the number of looks. *ρ* represents the coherence of the SAR image pair. It is related to the SNR, which is [24]:

(33) $$\rho =\frac{1}{1+SNR^{-1}}$$

**Figure 12:**
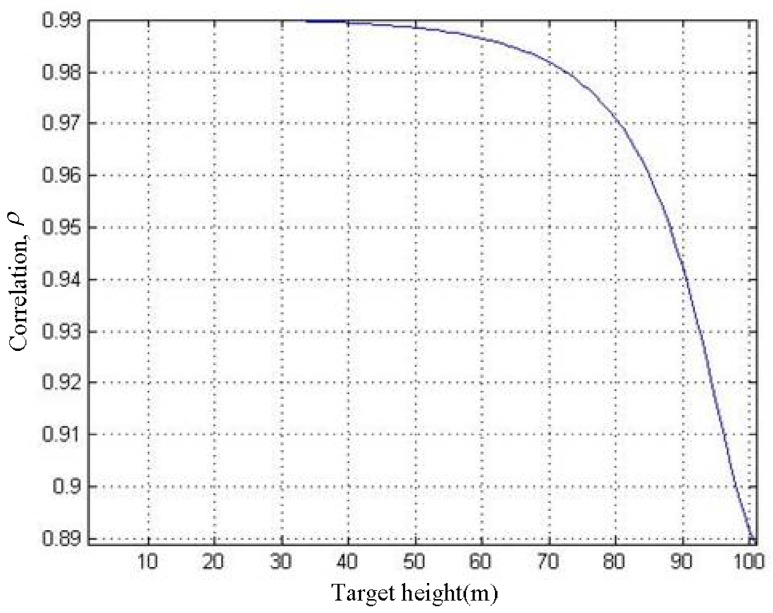
*h*–*ρ* curve.

With reference to Equations (31) and (33), the *h*–*ρ* curve can be obtained, as shown in Figure 12.

**Figure 13:**
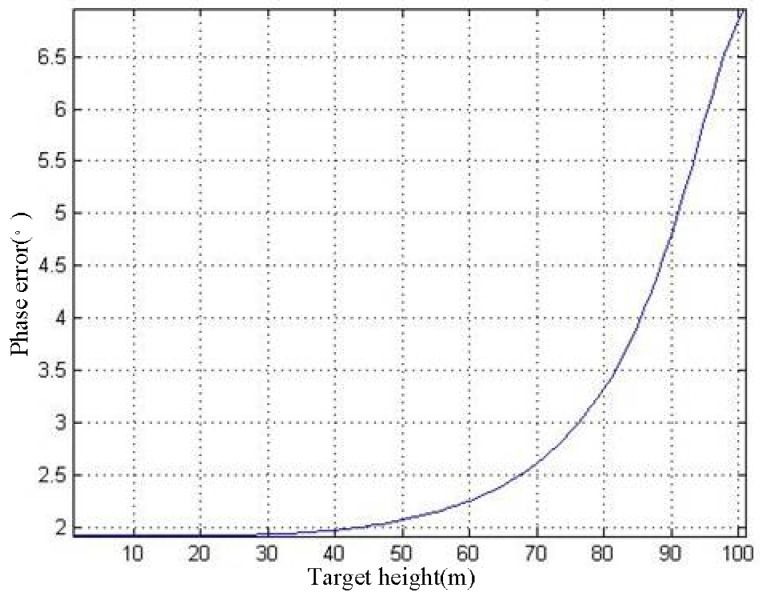
*h*–*σ*_ps_ curve.

It can be seen in Figure 12 that as *h* increases, *ρ* gradually decreases. When *h* = 100 m, *ρ* decreases to 0.89. We use the 3 × 3 filter window for phase filtering, thus *NL* = 9. According to Equation (32) and Figure 12, the *h*–*σ*_ps_ curve can be acquired, as shown in Figure 13.

As shown in Figure 13, as *h* increases, *σ*_ps_ gradually rises. When *h* = 100 m, the *σ*_ps_ rises to 6.96.

#### 5.1.2. Phase Error due to the Difference in Phase Distortion of the SAR Images

**Figure 14:**
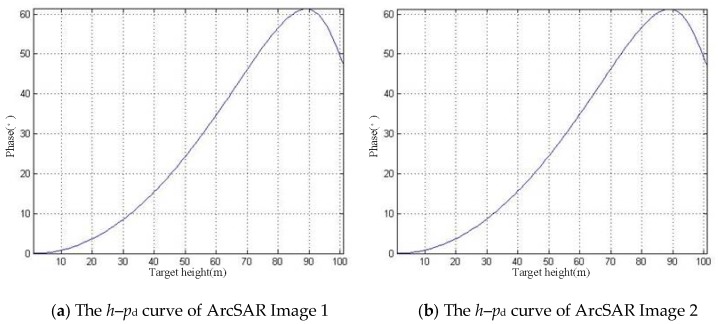
The *h*–*p*_d_ curves.

The defocusing can cause phase distortion in SAR images. If there is the difference in phase distortion of the SAR image pair used for interference, the phase error will be introduced during the interference process. In this section, we analyze this kind of phase error. We define the distortion phase as *p*_d_. The *h*–*p*_d_ curves of the SAR image pair are shown in Figure 14.

**Figure 15:**
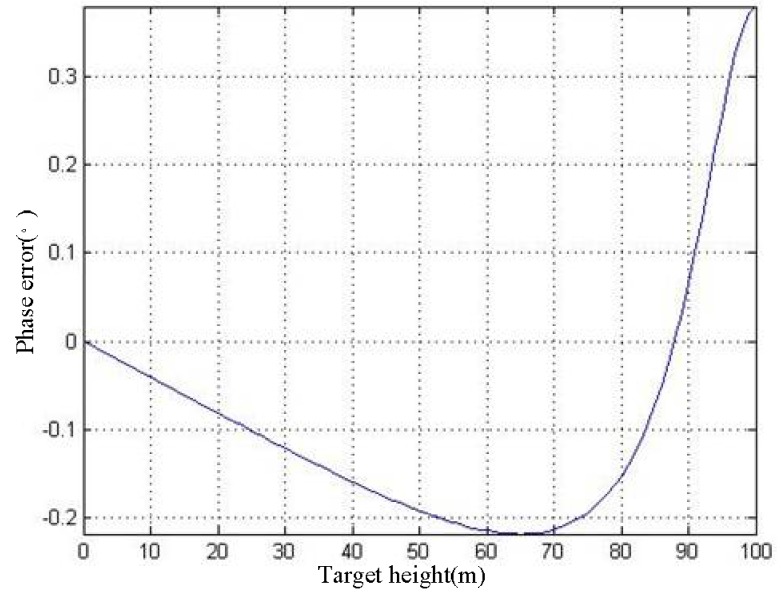
*h*–*σ*_pd_ curve.

It can be found that the phase distortions of the above SAR image pair are similar, which means that most of the distortion phase will be offset during the interference process. Therefore, the phase error introduced by the process of interference is small, as shown in Figure 15.

*σ*_pd_ represents the phase error due to the difference in phase distortion of the SAR image pair.

#### 5.1.3. The Effects of Phase Errors on DEM Accuracy

With reference to Equations (25), (26) and (28), we can derive the relationship between DEM accuracy *σ_h_* and the above two types of phase error:

(34) $$\sigma_{h}=\frac{\lambda R_{\mathrm{sp}}}{4\pi \Delta z}\left(\left|\sigma_{\mathrm{ps}}\right|+\left|\sigma_{\mathrm{pd}}\right|\right)$$

**Figure 16:**
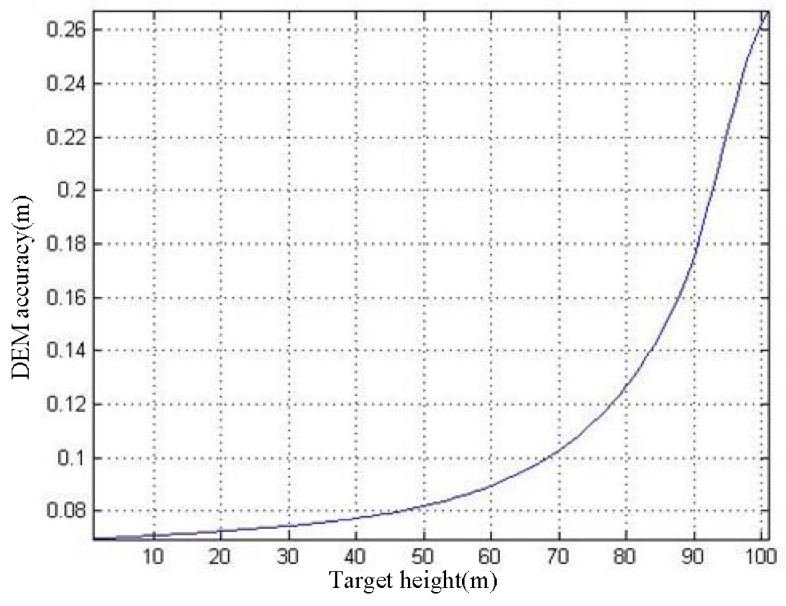
*h*–*σ_h_* curve.

According to Equation (34), Figure 13 and Figure 15, the *h*–*σ_h_* curve can be obtained, as shown in Figure 16.

Referring to the conclusion of Section 3.2, when the *R*_sp_ = 300 m, the height difference threshold is 38.26 m. Therefore, if the accuracy of the DEM for assisting imaging is better than 38.26 m, the proposed imaging method can obtain high-precision ArcSAR imaging results. The curve in Figure 16 shows that the DEM accuracy does not exceed 0.5 m, which is much smaller than the height difference threshold. Thus, we can draw the following conclusion: Even if the SAR images used for interference appears defocused, the obtained DEM can still be used to assist ArcSAR imaging and acquire high precision image.

### 5.2. Deformation Monitoring Accuracy Analysis

We utilize the DEM obtained by interferometric ArcSAR to assist imaging, which can significantly improve the imaging quality. The improvement of image quality also helps to improve the deformation monitoring accuracy. In this part, the contribution of the proposed method to the improvement of deformation monitoring accuracy is analyzed. As a comparison, the impact of traditional ArcSAR imaging algorithm (the imaging method with the image on the reference plane) on the deformation monitoring accuracy is also discussed. With reference to Section 5.1, defocusing causes two types of phase error. They will affect the accuracy of deformation monitoring. We define the deformation monitoring accuracy as *σ*_d_, which can be expressed as [7]:

(35) $$\sigma_{d}=\frac{\lambda }{4\pi }\left(\left|\sigma_{\mathrm{ps}}\right|+\left|\sigma_{\mathrm{pd}}\right|\right)$$

**Figure 17:**
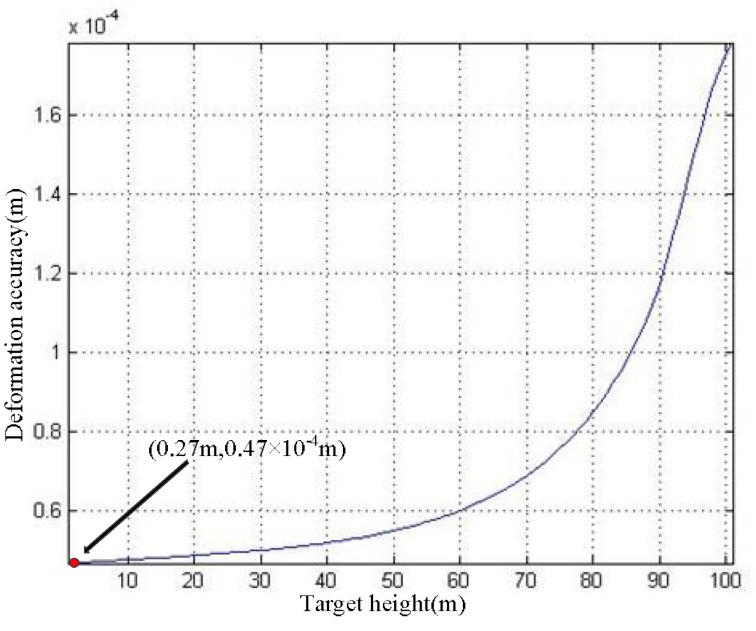
*h*–*σ*_d_ curve.

According to Equation (35), Figure 13 and Figure 15, the *h*–*σ*_d_ curve can be acquired, as shown in Figure 17.

For the convenience of analysis, we take the deformation monitoring accuracy at *h* = 100 m as the example. Referring to the curve in Figure 17, the deformation monitoring accuracy of the traditional ArcSAR imaging algorithm is 1.78 × 10^−4^ m. If we use the proposed imaging method, the target in the scenes will be imaged on its real position. According to Figure 16, the DEM accuracy at h = 100 m is *σ_h_*(100 m) = 0.27 m. Therefore, the deformation monitoring accuracy of the proposed method can reach to *σ*_d_(0.27 m) = 0.47 × 10^−4^ m, as marked in Figure 17. Through the above analysis, we can conclude that, compared with the traditional ArcSAR imaging algorithm, the imaging method proposed in this paper can effectively improve the deformation monitoring accuracy.

## 6. Experiment

We used a distributed scenes imaging experiment of ArcSAR to verify the effectiveness of the proposed high-precision imaging method. The experiment contained two parts. First, we used the ArcSAR images of the distributed scenes for interference to extract the DEM image of the scenes. Then, the extracted DEM image was transformed from the slant range to the ground range using the polar coordinate transformation method proposed in Section 4.2. Second, we performed the distributed scenes ArcSAR imaging simulation experiment. The DEM image obtained in Experiment 1 was used for assisted imaging.

### 6.1. The Simulation Experiment of Interferometric ArcSAR Extraction DEM

**Figure 18:**
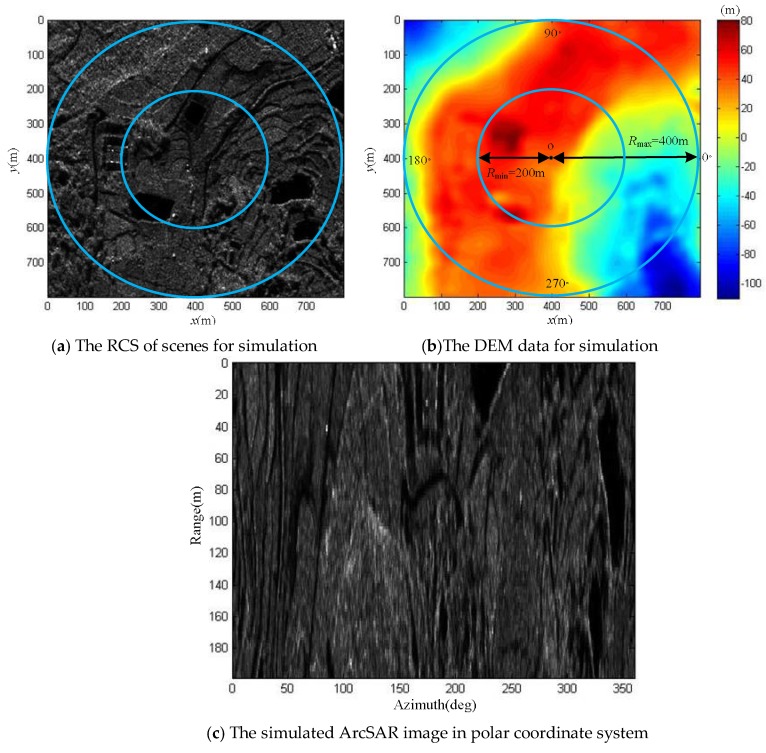
The RCS scenes for simulation, the DEM data for simulation and the simulated ArcSAR image in polar coordinate system.

Based on the geometric model and working mode of the ArcSAR system, we uses the existing RCS scenes and DEM data to simulate the ArcSAR image on the slant range. To facilitate subsequent experiment, the simulated ArcSAR image is shown in the polar coordinate system in Figure 18. The necessary parameters are given in Table 2.

*R*_max_ represents the maximum slant range from the imaging scenes to the ArcSAR system and *R*_min_ represents the minimum slant range from the imaging scenes to the ArcSAR system. They are marked in Figure 18b. The point o in Figure 18b is the rotation center. We defined the height of rotation plane to be 0 m. In Figure 18c, the horizontal axis represents the rotation angle of the ArcSAR system, and the vertical axis is the interval from *R*_min_ to *R*_max_.

**Figure 19:**
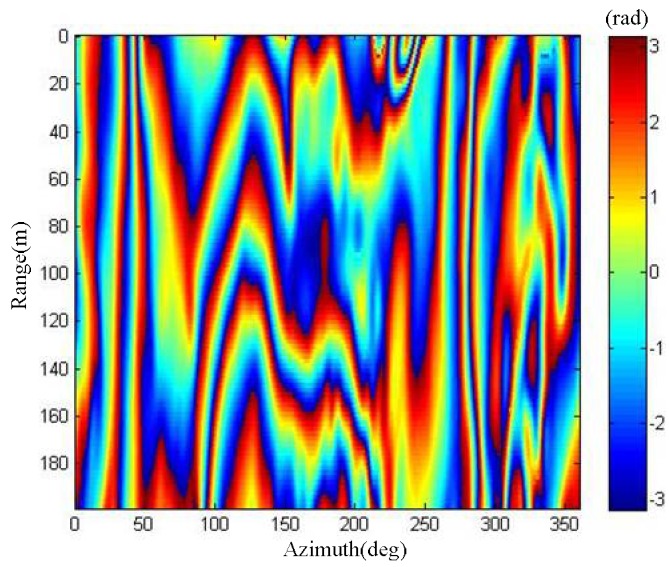
Interferometric phase image of phase wrapping.

The ArcSAR imaging result in Figure 18c was considered to be the main complex image for interference. The height baseline ∆*z* was 0.2 m. We simulated the sub SAR complex image for interference. The interferometric phase can be obtained by interference with the main SAR complex image and the sub SAR complex image. The interferometric phase image is shown in Figure 19.

**Figure 20:**
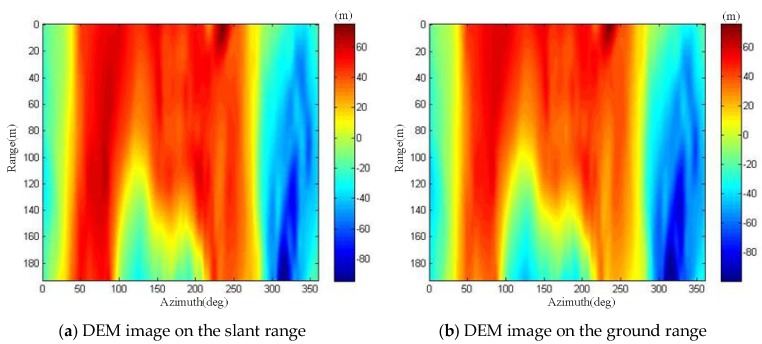
Interferometric ArcSAR extraction the DEM image of scenes.

The measured interferometric phase value shown in Figure 19 was modulated by 2π, ranging from −π to π, and there was an ambiguity of many cycles in the interferometric phase value. Thus, it was necessary to perform phase unwrapping on the interferometric phase image. The branch cut method was applied to interferometric phase unwrapping [25,26]. In addition, to reduce the complexity of phase unwrapping, we also removed the flat phase of the interferometric phase. After the flat phase removal and phase unwrapping operations, the interferometric phase image could be used for DEM inversion. The DEM image obtained by the interferometric phase inversion is the slant range image, as shown in Figure 20a. We utilized the proposed polar coordinate transformation method to transform it to the ground range. The DEM image on the ground range is shown in Figure 20b.

We also calculated the mean square error (MSE) of the DEM image in Figure 20b with the existing DEM data. The result of the MSE was only 1.69. It can be seen that the interferometric ArcSAR could acquire high-precision DEM image of the scenes.

### 6.2. Distributed Scenes Imaging Simulation Experiment Verification the DEM-Assisted High Precision Imaging Method for ArcSAR

**Figure 21:**
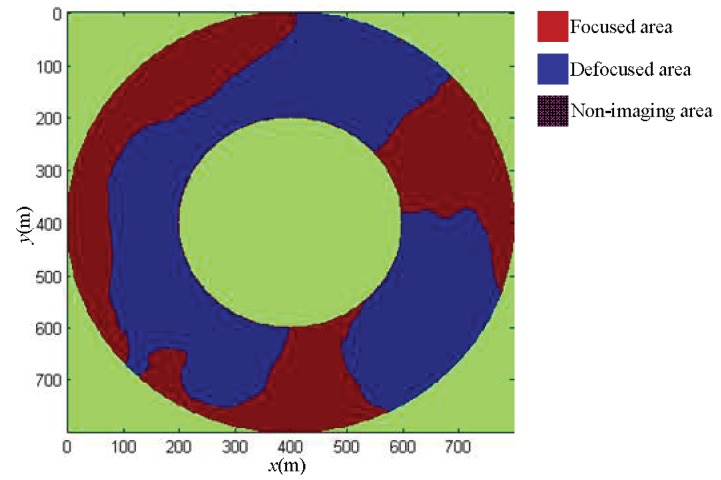
The defocused area and focused area of imaging result on the reference plane.

We used the distributed scenes imaging simulation experiment to image the scenes given in Experiment 1. The necessary parameters are shown in Table 2. The DEM image acquired in Experiment 1 was applied to assist ArcSAR imaging. In addition, we also used the traditional imaging method of ArcSAR to image the distributed scenes on the reference plane as the comparison. The height of the reference plane was 0 m. Based on Equations (17)–(19) in Section 3.2, we found the defocused area and focused area of the distributed scenes imaging results on the reference plane, as shown in Figure 21. 

**Figure 22:**
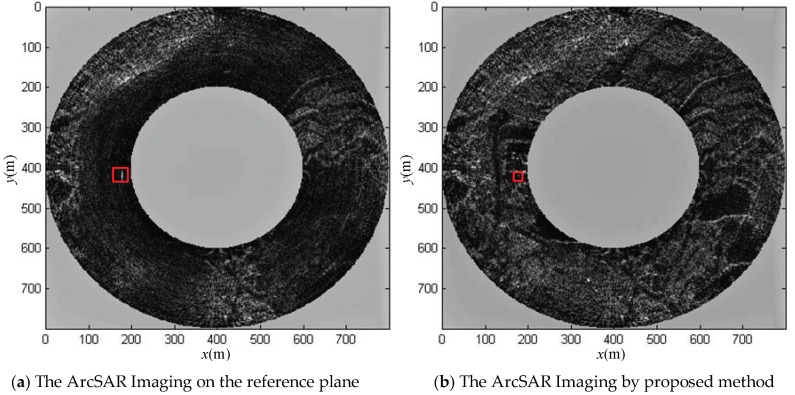
The distributed scenes imaging simulation results of ArcSAR in Cartesian coordinate system by traditional method and proposed method.

The imaging results by the two imaging methods in Cartesian coordinate system are shown in Figure 22.

**Figure 23:**
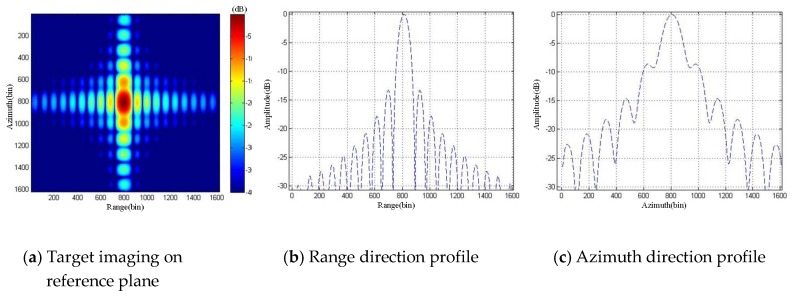
Imaging simulation results of the strong scattering target on the reference plane.

**Figure 24:**
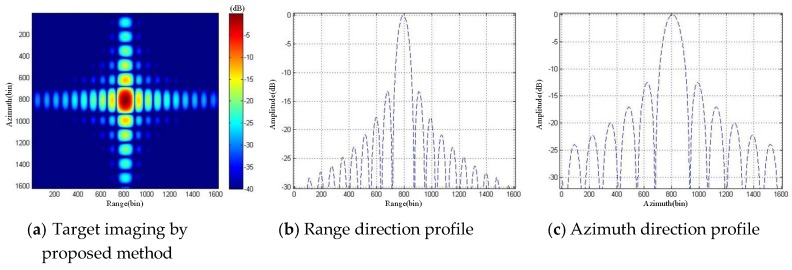
Imaging simulation results of the strong scattering target by proposed method.

It can be seen that the ArcSAR imaging result of the scenes on the reference plane showed severe defocusing, and the ArcSAR image obtained by the proposed imaging method was not defocused. To further analyze the imaging accuracy of the proposed imaging method, we set a strong scattering target in the scenes and analyzed the quality of its imaging result. Its imaging result is marked in Figure 22. We sliced the imaging result of the target and up sampled it 20 times, as shown in Figure 23 and Figure 24. The imaging quality parameters of the strong scattering are shown in Table 3.

According to Equations (17)–(19) in Section 3.2, we calculated that the height difference threshold of the strong scattering target was 28.28 m. The height of the strong scattering target in Figure 22 was 36.82 m, which exceeded height difference threshold. Therefore, its imaging result on the reference plane showed severe defocusing, as shown in Figure 23. The imaging result of the strong scattering target obtained by the proposed high-precision imaging method was not defocused, as shown in Figure 24. Besides, it can be seen from the analysis results of the imaging quality in Table 3 that the proposed imaging method could effectively improve the quality of ArcSAR image and achieve the high-precision imaging.

## 7. Conclusions

In this paper, an interferometric DEM-assisted high precision imaging method for ArcSAR is proposed. The proposed method applies the interferometric ArcSAR to extract the DEM of scenes. The extracted DEM is utilized to assist ArcSAR imaging. This operation enables the target in the scenes image on its actual height. The proposed imaging method does not rely on external DEM data. Compared with the traditional ArcSAR algorithm imaged on the reference plane, the proposed method can effectively improve the accuracy of ArcSAR imaging. 

## Figures and Tables

**Table 1:** The ArcSAR system parameters.

r (m)	*θ*_bw_ (rad)	*θ* (rad)	*λ* (mm)	*R*_sp_ (m)	*Br* (MHz)
1	π/3	π/3	17.50	300	150

**Table 2:** Experiment parameters.

*r* (m)	*θ*_bw_ (rad)	*θ* (rad)	*λ* (mm)	*Br* (MHz)	*R*_max_ (m)	*R*_min_ (m)
1	π/3	2π	17.50	150	400	200

**Table 3:** The quality parameters of the strong scattering target imaging result.

Parameters	Imaging on Reference Plane	Imaging by Proposed Method
Coordinates (m,deg)	(220.91,185.19)	(220.91,185.19)
Height (m)	36.82	36.82
Range direction resolution (m)	1.00	1.00
Range direction PSLR (dB)	−13.21	−13.22
Azimuth direction resolution (m)	1.92	1.91
Azimuth direction PSLR (dB)	−8.63	−12.57
